# MiR-125a-3p timely inhibits oligodendroglial maturation and is pathologically up-regulated in human multiple sclerosis

**DOI:** 10.1038/srep34503

**Published:** 2016-10-04

**Authors:** Davide Lecca, Davide Marangon, Giusy T. Coppolino, Aida Menéndez Méndez, Annamaria Finardi, Gloria Dalla Costa, Vittorio Martinelli, Roberto Furlan, Maria P. Abbracchio

**Affiliations:** 1 Laboratory of Molecular and Cellular Pharmacology of the Purinergic Transmission, Dipartimento di Scienze Farmacologiche e Biomolecolari, Università degli Studi di Milano, Milan, 20133, Italy; 2 Departamento de Bioquímica y Biología Molecular IV, Universidad Complutense de Madrid, 28040, Spain; 3 Institute of Experimental Neurology (INSpe), Division of Neuroscience, San Raffaele Scientific Institute, Milan, 20132, Italy

## Abstract

In the mature central nervous system (CNS), oligodendrocytes provide support and insulation to axons thanks to the production of a myelin sheath. During their maturation to myelinating cells, oligodendroglial precursors (OPCs) follow a very precise differentiation program, which is finely orchestrated by transcription factors, epigenetic factors and microRNAs (miRNAs), a class of small non-coding RNAs involved in post-transcriptional regulation. Any alterations in this program can potentially contribute to dysregulated myelination, impaired remyelination and neurodegenerative conditions, as it happens in multiple sclerosis (MS). Here, we identify miR-125a-3p, a developmentally regulated miRNA, as a new actor of oligodendroglial maturation, that, in the mammalian CNS regulates the expression of myelin genes by simultaneously acting on several of its already validated targets. In cultured OPCs, over-expression of miR-125a-3p by mimic treatment impairs while its inhibition with an antago-miR stimulates oligodendroglial maturation. Moreover, we show that miR-125a-3p levels are abnormally high in the cerebrospinal fluid of MS patients bearing active demyelinating lesions, suggesting that its pathological upregulation may contribute to MS development, at least in part by blockade of OPC differentiation leading to impaired repair of demyelinated lesions.

MicroRNAs (miRNAs) are a class of small, highly conserved, non-coding RNA molecules (approximately 21–25 nucleotides in length) that control gene expression by binding to complementary sequences in the 3′ untranslated regions of their target messenger RNAs, therefore resulting in either translational repression or degradation^1^. During their biogenesis, primary miRNAs (pri-miRNAs) generate double stranded miRNA consisting of two arms, namely 3p and 5p, both of which can act as active mature miRNAs with distinct biological functions and targets^2^,^3^. These abundant, endogenous, post-transcriptional regulators are involved in important processes such as development, metabolism, cell proliferation, differentiation and death^4^. The power of this regulatory system relies on the unique ability of miRNAs to guide cellular processes through precise titration of gene expression, and on the possibility for a single miRNA to control a large number of gene products. Thus, the action of a single miRNA can lead to a cumulative reduction in the expression of multiple components of one specific functional network, and several miRNAs, transcribed in clusters, may cooperatively target various mRNAs whose protein products are part of the same molecular pathway^5^,^6^.

Among the most important miRNAs, the miR-125 family (miR-125a and miR-125b) has been reported to be implicated as either repressors or promoters in a variety of carcinomas and other diseases^7^. Recently, it has been also demonstrated that miRNAs belonging to this family are brain-enriched^8^ and they exert important roles in nervous system development. Both miR-125a and miR-125b have been shown to induce the irreversible commitment of human pluripotent stem cells to the neural lineage^9^ and glial progenitors to undertake astroglial differentiation^10^. Despite the presence of miR-125a (in both its 5p and 3p strands) in other neural cells such as oligodendrocytes has been already demonstrated^11^,^12^, its role in these cells has never been investigated.

During differentiation, oligodendroglial precursor cells (OPCs) progressively increase their arborisation, make contacts with surrounding axons and finally produce myelin, an insulating substance wrapping axons and responsible for the correct transmission of the nervous impulse^13^. During development, OPCs arise from the ventricular zone in the embryonic brain and spinal cord, they proliferate and migrate through the whole parenchyma, and then differentiate to mature cells^14^. A subset of OPCs remains active throughout life, periodically differentiating to mature cells, thus assuring continuous myelin replacement. Each stage of differentiation can be identified based on the expression of typical oligodendroglial markers, among which MBP (myelin basic protein) is typically expressed in mature/myelinating oligodendrocytes^13^.

The whole differentiative program of OPCs is strictly regulated by genic and epigenetic mechanisms, including miRNAs^15^. Interestingly, *in vitro* interaction studies performed in HEK293 cells demonstrated that miR-125a-3p is able to bind and inhibit the expression of Fyn, a tyrosine kinase^16^ which has been implicated in axon-glial signal transduction and in cellular processes required for the maturation of oligodendrocytes^17^. Other important players in cytoskeletal functions and glial cell differentiation, such as the small GTPase RhoA, p38 and neuregulin 1 (Nrg-1), have been identified as direct targets of this miRNA^18^,^19^,^20^, suggesting that miR-125a-3p could participate in the regulation of oligodendroglial functions.

Here we show, for the first time, a role for miR-125a-3p in oligodendroglial differentiation. In OPCs, its overexpression strongly delayed oligodendroglial maturation likely due to a silencing effect on different actors involved in the same molecular pathways leading to myelination. Of high interest for the pathophysiology of a demyelinating disease like multiple sclerosis (MS), levels of miR-125a-3p were altered in the cerebrospinal fluid of MS patients, suggesting a link with the disease, but also a possible contribution to defective remyelination abilities of these patients.

## Results

### MiR-125a-3p potentially targets players involved in oligodendrocyte differentiation

**Figure 1:**
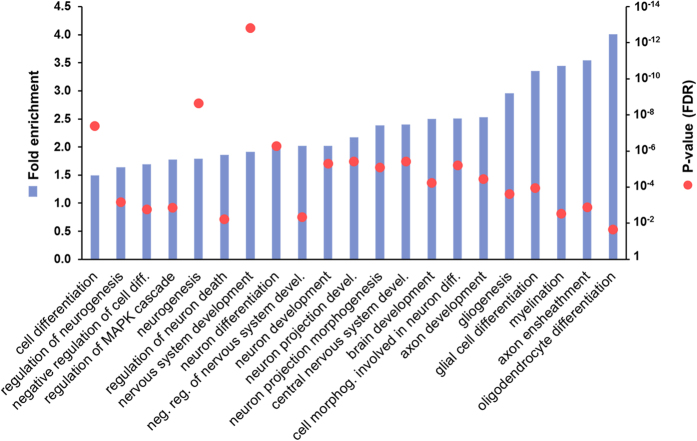
Gene Ontology biological processes (GO BPs) related to oligodendrocyte development are enriched in miR-125a-3p targets. Histograms in blue show the significant GO BPs enriched in miR-125a-3p target transcripts (fold enrichment compared to expected value = 1; see left axis). Red dots represent p-value (with FDR correction) of the prediction for each GO BP (see right axis; log_10_ scale).

To evaluate the potential relevance of miR-125a-3p in oligodendrocyte differentiation and myelination, we performed in silico analysis on mouse databases. First, we used MyMIR, a system that performs meta-predictions based on integration, filtering and re-ranking of outputs produced by different miRNA databases and we obtained a list of 1,673 unbiased putative target transcripts of this miRNA. Then, by using STRING, a Gene Ontology (GO) based tool, we found that these targets were significantly clustered in 485 GO biological processes (GO BPs; Supplementary Table S1), suggesting that miR-125a-3p can take part in their regulation. In agreement with previous findings that miR-125a-3p is a brain-enriched miRNA, we highlighted that many GO BPs include nervous system development, neurogenesis, glial cell differentiation, myelination, and oligodendrocyte differentiation (Fig. 1). For all of them, we calculated and reported the fold enrichment in miR-125a-3p targets (see equation (1) in Methods) and we found that the BPs related to oligodendrocyte differentiation have a higher enrichment among all the CNS-related BPs, strongly supporting our hypothesis.

### Time-regulated expression of MiR-125a-3p in cultured OPCs

To assess the expression of miR-125a-3p in the CNS, by means of real-time PCR we measured its levels in several rodent and human tissues and cells. Our analysis showed that indeed, miR-125a-3p it is more abundantly present in the CNS of both rat and human.

**Figure 2:**
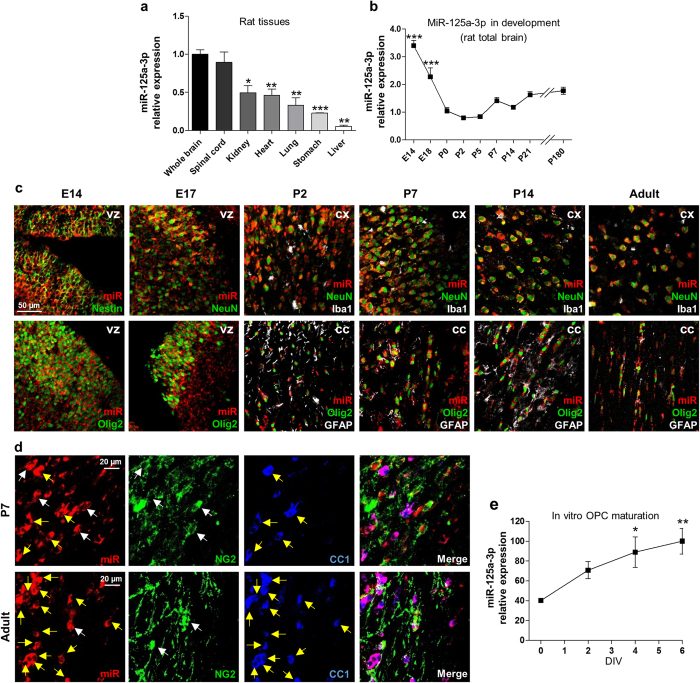
Expression of miR-125a-3p in physiological conditions. (**a**) miR-125a-3p relative expression was analyzed using Taqman assay in different rat tissues and compared to level measured in brain. Neural tissues showed higher expression with respect to other tissues. Data were expressed as mean ± SEM, n = 3; two-tailed unpaired t test *p < 0.05, **p < 0.01, ***p < 0.001 vs. total brain. (**b**) The levels of miR-125a-3p were analyzed during rat brain development and normalized to the levels at P0. Data were expressed as mean ± SEM, n = 4 animals for each time-point; one-way ANOVA with Dunnett’s post-test ***p < 0.001 vs. P0. (**c**) *In situ* hybridization of miR-125a-3p (red fluorescence) in combination with different lineage markers during rat brain development from embryonic day 14 (E14) to adulthood. In embryonic brain the ventricular zone (vz) was chosen. In postnatal brain neurons stained with NeuN were shown in cerebral cortex (cx), whereas Olig2^+^ oligodendrocytes were shown in corpus callosum (CC). Iba1 and GFAP were shown in triple stainings (white pseudocolor) to identify microglia and astrocytes, respectively. (**d**) *In situ* hybridization of miR-125a-3p (in red) in double staining with both NG2 (early OPC marker; in green), and CC1 (marker of mature oligodendrocytes; in blue). Micrographs show representative pictures of corpus callosum of P7 rats (upper panels) and adult rats (lower panels). White arrows highlight co-localization of miR with NG2, while yellow arrows refer to co-localization with CC1. (**e**) Time-course of mir-125a-3p expression in OPCs was analyzed in differentiating conditions and normalized to the levels in undifferentiated OPC (day 0). MiR-125a-3p levels progressively raised, reaching a 2.5-fold increase after 6 days in culture. Data were expressed as mean ± SEM, n = 6; one-way ANOVA with Dunnett’s post-test; *p < 0.05, **p < 0.01 vs. day 0.

In adult rat, the highest expression was found in both total brain and spinal cord, whereas very low levels were found in kidney, heart, lung, stomach and liver (Fig. 2a). We performed the same analysis on human samples, and, among the analysed tissues, miR-125a-3p was found mainly expressed in brain tissues, with a significant enrichment in frontal cortex compared to whole brain and also in brain stem and corpus striatum (Supplementary Fig. S1). In line with the rodent data, lower expression levels were found in kidney and liver, confirming the brain enrichment of this miRNA. Then, we asked whether miR-125a-3p is regulated in rat developing brain. To this aim, we collected RNA from total rat brain at different ages starting from embryonic day 14 (E14) to adulthood (6 months of age). Levels of miRNA were already high at E14, gradually declined up to postnatal day 0 (P0), remained low after birth up to P5, increased immediately before myelination onset and then remained mostly stable until adulthood (Fig. 2b).

To evaluate the distribution of this miRNA in the different neural cell types during brain development, we took advantage of *in situ* hybridization (ISH) in combination with immunohistochemistry for cell markers, namely Nestin for multipotent neural stem cells, NeuN for neurons, GFAP for astrocytes, Olig2 for oligodendrocytes and Iba1 for microglia. In embryonic brain, miR-125a-3p was found widespread in the whole parenchyma with a higher signal in the ventricular zone (Fig. 2c), where it mostly co-localized with Olig2, which, at this stage, labels a pool of precursor cells that will give rise to both OPCs and neurons^21^, and to a lower extent with Nestin. In postnatal brain, the expression of miR-125a-3p is restricted to neurons, both in cortex and striatum, and oligodendrocytes (Fig. 2c), whereas only a few cells also expressed GFAP and no apparent co-localization was found with microglia.

Then, we focused on oligodendrocytes, and evaluated whether miR-125a-3p is preferentially expressed in early OPCs expressing the proteoglycan NG2, or in more mature CC1^+^ oligodendrocytes. To this aim, we performed a triple immunofluorescence staining using the same ISH technique described above for miR-125a-3p in parallel with anti-NG2 and anti-CC1 antibodies in both postnatal (P7) and adult brains, in particular, corpus callosum, where a large number of oligodendrocytes could be analysed. In postnatal brain, some cells already expressed CC1 and virtually all of them were also decorated with the probe for miR-125a-3p (Fig. 2d). As expected, the number of CC1^+^ cells in the adult brain was much higher, but also in this case, virtually all cells expressed miR-125a-3p. Both in P7 and adult brains, co-localization between miR-125a-3p and NG2 was also found, but only in a subpopulation of cells, with a further trend toward a decrease in the adult, suggesting that miR-125a-3p serves a more critical function in mature oligodendrocytes.

Then, to assess if the expression timing of miR-125a-3p indeed changes during oligodendrocyte maturation, we used cultured OPCs to measure its expression during their *in vitro* differentiation. Interestingly, the levels of miR-125a-3p progressively increased in OPCs kept in cultures under differentiating conditions with the triiodothyronine hormone (T3), reaching a 2.5-fold increase after 6 days (Fig. 2e) compared to the initial proliferating conditions (day 0, in the presence of growth factors and in the absence of T3).

Globally, the *in vivo* and *in vitro* data suggest multiple developmental roles for miR-125a-3p, that, during embryonic life, is expressed at high levels in neural undifferentiated precursors likely to prevent their untimely commitment, and that, within cells of the oligodendroglial lineage, it could crucially regulate maturation by repressing early transcripts important for the maintenance of OPCs at an undifferentiated state.

Since we were interested in myelination, we investigated more in detail the possible role of miR-125a-3p during physiological differentiation of OPCs to mature oligodendrocytes.

### Over-expression of miR-125a-3p by mimic treatment impairs while its inhibition with an antago-miR stimulates oligodendroglial maturation

**Figure 3:**
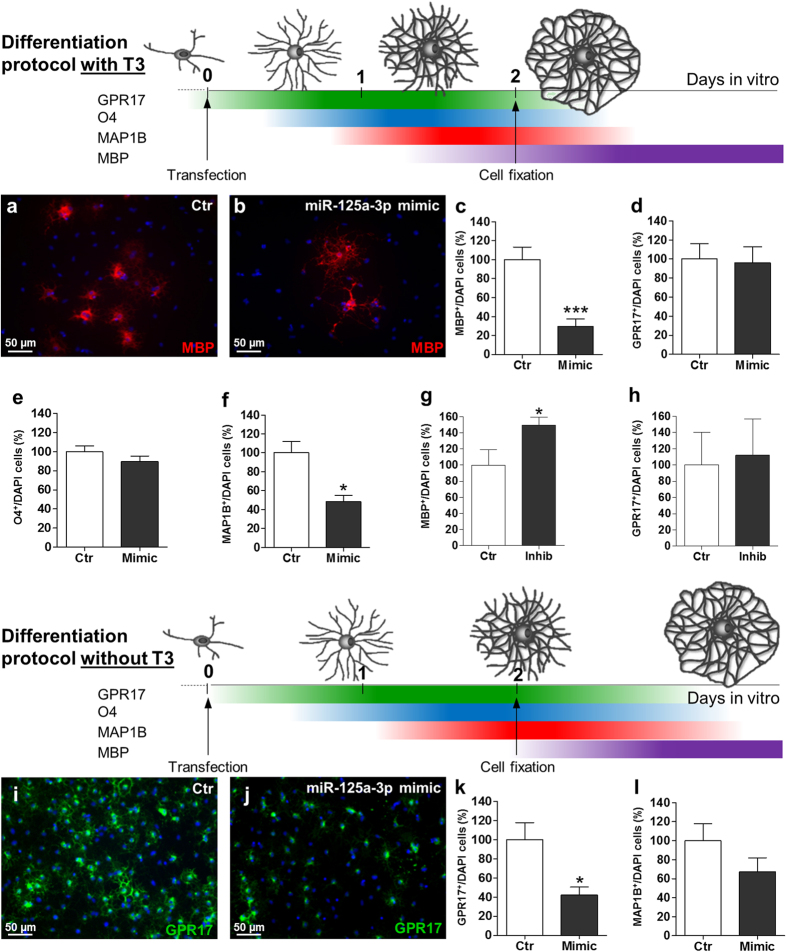
Effect of over-expression or inhibition of miR-125a-3p during oligodendroglial differentiation. To evaluate the time-dependent effects of either the overexpression or the silencing of miR-125a-3p on OPC maturation 4 different markers of progressive differentiation stages were analysed by immunocytochemistry, GPR17 and O4 (which label pre-oligodendrocytes), MAP1B (which labels a slight more advanced stage) and MBP (which labels terminally differentiated cells). Cells were stained for the selected markers 48 h after transfection of miR-125a-3p mimic or inhibitor, in the presence or in the absence of the T3 hormone. (**a–h**) The drawing represents a schematic timeline of the fast differentiation protocol in the presence of T3, showing the presented markers. (**a,b**) Representative micrographs of MBP^+^ oligodendrocytes after transfection of miR-125a-3p mimic in presence of T3 were shown. (**c–f**) Histograms show the changes in the number of cells positive for MBP (n = 10), GPR17 (n = 8), O4 (n = 2) and MAP1B (n = 3) with respect to negative controls. Data were expressed as mean ± SEM, two-tailed unpaired t test *p < 0.05, ***p < 0.001 vs. negative control. (**g,h**) Transfection of OPC with the inhibitor of miR-125a-3p (in presence of T3) led to a significant increase in the number of MBP^+^ (n = 4; p-value = 0.0376), but not GPR17^+^ (n = 7) cells. Data were expressed as mean ± SEM, two-tailed unpaired t test *p < 0.05 vs. negative control. (**i–l**) A second differentiation protocol in the absence of T3 (slower protocol) was used. Also in this case, the drawing represents a schematic timeline showing the presented markers. (**i,j**) Representative micrographs of GPR17^+^ oligodendrocytes after transfection of miR-125a-3p mimic in absence of T3 were shown. (**k,l**) Histograms show a considerable decrease in the number of GPR17^+^ (n = 7) and MAP1B^+^ (n = 3) cells after transfection with the mimic in the absence of T3 with respect to the negative control. Data were expressed as mean ± SEM, two-tailed unpaired t test *p < 0.05 vs. negative control.

When OPCs are cultured in the absence of growth factors and in the presence of the T3 hormone, they undergo rapid *in vitro* differentiation, whose phases can be easily monitored by staining cells for progressively more mature oligodendrocyte markers (drawing in Fig. 3).

To evaluate the possible role of miR-125a-3p in OPC differentiation, we overexpressed it by transfecting cells with a specific miR-125a-3p mimic the same day of growth factors withdrawal (corresponding to day 0 in Fig. 2e) when the endogenous levels of miR-125a-3p were low. Then, we performed immunofluorescence stainings for the myelin basic protein (MBP), a typical marker of mature oligodendrocytes and counted cells in mimic-transfected cultures and in control conditions (i.e., cells transfected with the corresponding negative mimic). Two days after miR-125a-3p mimic transfection, the number of MBP^+^ cells was strongly reduced compared to controls, suggesting an effect on terminal maturation (Fig. 3a–c). Since MBP is not present in the list of miR-125a-3p putative targets obtained from MyMIR, we reasoned that miR-125a-3p inhibitory effect is likely exerted on upstream players in the pathway culminating with OPC maturation and MBP expression. Thus, we also counted cells positive for earlier oligodendroglial players, such as, sequentially, GPR17 (a marker of early precursors and intermediate immature oligodendrocytes)^22^,^23^, O4 (a marker of immature oligodendrocytes)^24^, and the microtubule associated protein MAP1B^25^ (that labels a slightly more advanced differentiation stage compared to O4, see drawing in Fig. 3). While the number of cells positive for GPR17 and O4 were unaltered (Fig. 3d,e), the number of MAP1B^+^ cells was reduced by approximately the 50% after mimic miR-125a-3p treatment (Fig. 3f), suggesting inactivation of transcripts specifically involved in the progression between the O4 and the MAP1B stage. To confirm the importance of miR-125a-3p in OPC maturation, in a parallel experiment, OPCs maintained under the same culture conditions were transfected with a hairpin inhibitor RNA that specifically inactivates miR-125a-3p. A marked increase in the number of MBP^+^ cells was found, yet with no significant effect on the number of GPR17^+^ cells (Fig. 3g,h). Globally, these data suggest that miR-125a-3p exerts a silencing effect on transcripts typical of OPC differentiation stages that are upstream to MAP1B but downstream to GPR17 and O4.

Then, we wondered whether mimic overexpression in cultured OPCs maintained under slower differentiating conditions could also affect the expression of these earlier transcripts.

To test this hypothesis, we cultured OPCs in the absence of both growth factors and T3. Under these settings, OPCs did undergo spontaneous differentiation in culture, but did not reach terminal maturation within the experimental observation times, thus representing a useful paradigm for our purpose (see drawing in Fig. 3). As expected, under this culturing protocol, almost no MBP^+^ cells were detected; however, upon mimic overexpression, the number of both GPR17^+^ cells and of MAP1B^+^ cells were strongly reduced (of −60% and −35%, respectively, Fig. 3i–l) compared to controls. Representative micrographs of OPCs transfected with mimic and stained with the selected markers are shown in Supplementary Fig. S2.

These data demonstrate that if differentiation proceeds at a lower speed, miR-125a-3p can also act on early targets, whereas, if differentiation is faster, miRNA can only act on later transcripts that are closer to terminal maturation.

These data also suggest that the post-transcriptional regulation of genes involved in oligodendrocyte maturation vary depending on basal or stimulated conditions such as those occurring upon CNS damage, when recruitment and differentiation of OPCs is rapidly and markedly improved to sustain myelin repair^26^.

### MiR-125a-3p acts on multiple target transcripts involved in oligodendrocytes maturation

**Figure 4:**
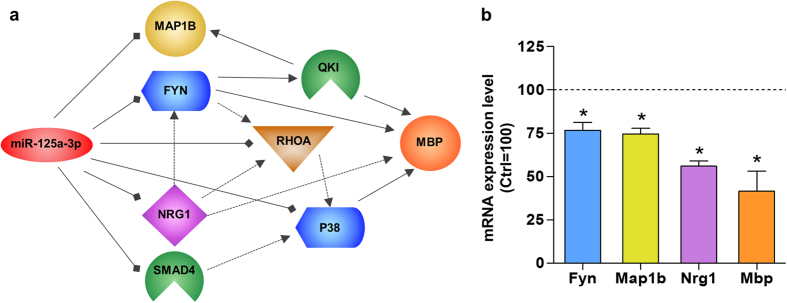
Model representing the connections between miR-125a-3p and MBP. (**a**) In this model we reconstructed a network showing how miR-125a-3p can influence MBP expression, starting from predicted (Map1b) and validated targets of this miRNA (Smad4, p38, RhoA, Nrg1 and Fyn). All these direct targets converge in a pathway leading to the maturation of oligodendrocytes. (**b**) To confirm this mechanism we performed qPCR experiments after miR-125a-3p over-expression in OPCs and we found that both Fyn (n = 3), Nrg1 (n = 3), Map1b (n = 3) and in turn Mbp (n = 5) mRNA were strongly down-regulated in these conditions. Data were expressed as mean ± SEM, two-tailed unpaired t test *p < 0.05 vs. negative control = 100.

Based on the current knowledge, a single miRNA can interact with hundreds of target transcripts with “imperfect matches”; this unique feature enables a fine titration of several players of the same pathway at the same time. Since, as mentioned above, Mbp is not a predicted target for miR-125a-3p, we used QIAGEN’s Ingenuity Pathway Analysis tool (IPA^®^, QIAGEN Redwood City, www.qiagen.com/ingenuity) to evaluate how alterations of already validated targets of the myelination pathway such as Smad4, Fyn, RhoA, p38 and Nrg1^9^,^16^,^18^,^19^,^20^ could potentially influence MBP expression, thus being responsible for the observed delay in oligodendroglial maturation. This analysis showed that not only Nrg1 and Fyn, but also RhoA and p38 (that are downstream targets of FYN signaling)^27^,^28^ can directly or indirectly promote MBP expression, suggesting that miR-125a-3p may limit oligodendroglial maturation through inhibition of some of these targets, if not all (Fig. 4a). The list of all the mentioned targets is reported in Table S2 along with their validated interactions. To confirm this hypothesis, we performed quantitative real time PCR experiments after miR-125a-3p over-expression in OPCs, and found that the expected reduction of Mbp mRNA was also accompanied by considerable downregulation of both Fyn, Nrg1 and Map1b (Fig. 4b).

### MiR-125a-3p is up-regulated in cerebrospinal fluid of MS patients

**Figure 5:**
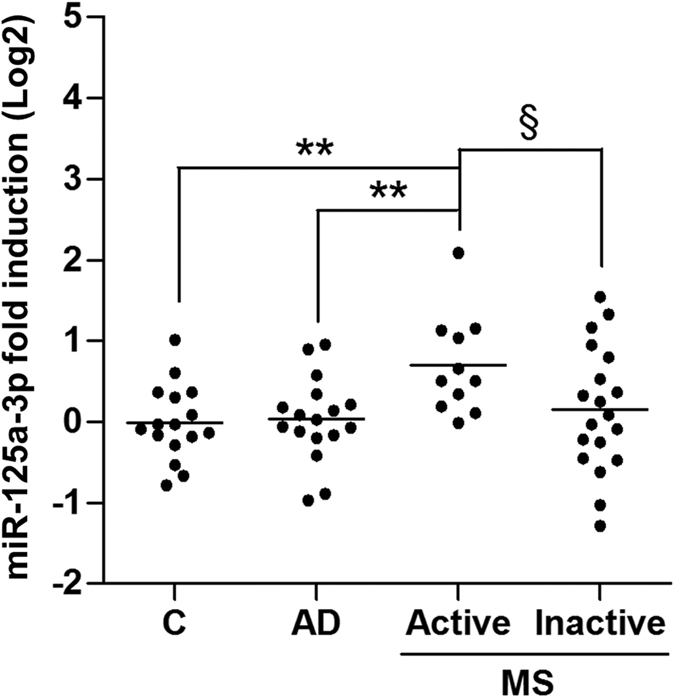
Expression of miR-125a-3p in the cerebrospinal fluid of MS patients. A significant increase in the relative expression of miR-125a-3p was found by RT-PCR in the cerebrospinal fluid of active MS patients (n = 11) compared to control (C; n = 13; p value = 0.004), Alzheimer (AD; n = 17; p value = 0.004) and inactive MS (n = 19; ^§^p = 0.0545) groups. Data were expressed as mean ± SEM; *p ≤ 0.05, **p < 0.01 Unpaired t test.

MiRNAs are stable in body fluids and their alterations in plasma and cerebrospinal-fluid (CSF) may represent novel biomarkers for the diagnosis and prognosis of human diseases. It has been proposed that circulating miRNAs are originated by passive leakage from broken cells, thus reflecting the disease state of injured tissues^29^. Since progressive loss of the ability of OPCs to generate mature oligodendrocytes is a well-known feature of both human MS and other demyelinating diseases^30^, we wondered whether miR-125a-3p were also altered in the CSF of MS patients. We thus measured miR-125a-3p levels in CSF samples from 30 MS patients (28 patients with the relapsing-remitting and 2 with the secondary-progressive form), 11 of which had clinically or neuroradiologically active disease (active lesions) at the time of CSF withdrawal. We compared MS patients to a control group mainly encompassing non-Alzheimer dementias and normal pressure hydrocephalus patients, and to a pure Alzheimer’s patients group (AD) (the demographic data of patients are summarized in Table 1). Quantitative real-time PCR analysis revealed a significant upregulation (up to 4 fold) of miR-125a-3p in the active MS group compared to both healthy subjects, AD and inactive MS groups (Fig. 5; p = 0.004 vs. controls; p = 0.004 vs. AD; p = 0.0545 vs inactive MS; Unpaired t test). Applying One-way ANOVA to consider multiple comparisons, active MS was still significantly different from the control group (p = 0.02). Although these findings are obviously not sufficient for proposing miR-125a-3p as a biomarker for MS, our results indeed suggest its potential clinical relevance in this disease.

## Discussion

Several papers have described the miR-125 family (composed by miR-125a and 125b) as a mostly important one for its implication in cancer^7^. Only recently, the role of miR-125a has been investigated also under physiological settings, when a fine balance between proliferation and differentiation is responsible for cell lineage commitment. However, studies in the literature did not analyze separately the specific role of either the −5p and the −3p strand, since they often refer to miR-125a as the double-stranded precursor miRNA^9^,^10^, and this may be misleading. Despite previous studies demonstrating its presence in oligodendrocytes, with higher abundance of the −3p compared to the −5p strand^11^, no functional roles for this miRNA in these cells had been yet demonstrated. Here, we describe miR-125a-3p as a new regulator of oligodendroglial differentiation with potential roles in myelination and in defective remyelination. First, our gene ontology based study showed that several of the predicted target mRNAs of this miRNA are involved in nervous system development, with higher enrichment in glial cell differentiation, myelination, axon ensheathment and oligodendrocyte differentiation (up to 4-fold).

Then, we show that, both in rodents and humans, miR-125a-3p is more abundantly expressed in CNS with respect to peripheral tissues, with a higher expression in neurons and oligodendrocytes. Our expression studies during brain development showed very high levels of miR-125a-3p at embryonic day E14, mainly in neural precursors. This is in line with previous literature data demonstrating that miR-125a directly binds Smad4, a key regulator of pluripotent stem cell lineage commitment, thus potentiating early neural specification^9^. Moreover, during postnatal life, SMAD4 and its cascade inhibit oligodendrogenesis by inducing the Id and Hes genes and by repressing Olig1 and Olig2^31^. Interestingly, by means of *in situ* hybridization, we found that miR-125a-3p is expressed throughout post-natal age in Olig2^+^ oligodendrocytes, suggesting that, in these cells, it is necessary for silencing SMAD4, in order to promote the expression of these genes and subsequent oligodendroglial specification. Several miRNAs have been demonstrated to regulate the transition from neural stem cells to mature oligodendrocytes. Indeed, as OPCs differentiate, the levels of several miRNAs including miR−138, −219 and −338 increase and promote maturation through the inhibition of repressors of oligodendrocytes differentiation, such as Hes5, Sox6, Foxj3, and of genes that promote OPC proliferation, such as Fgfr2 and Pdgfrα^32^,^33^. In line with the hypothesis of the modulation of early targets, during *in vitro* OPC differentiation miR-125a-3p is progressively up-regulated (Fig. 2e). Almost all mature oligodendrocytes, positive for CC1 express miR-125a-3p, whereas only a subset of NG2^+^ cells express it (Fig. 2d), suggesting a role in the repression of early genes when oligodendrocytes become mature and/or myelinating.

The recent literature demonstrated that a single miRNA can regulate hundreds of transcripts, thus having a very broad array of functional consequences^34^. Thus, integration of the information on all the validated targets of a given miRNA can shed light on very complex regulatory networks similar to those of transcription factors. In this respect, our data on the role of miR-125a-3p on OPCs maturation under “strong” or “mild” differentiation paradigms unveil that myelination may be regulated in different ways even by the same miRNA depending upon distinct pathophysiological conditions. Specifically, to identify the targets of miR-125a-3p during OPC differentiation *in vitro*, we took advantage of 4 different markers that identify 4 distinct sequential stages of OPC differentiation, namely: GPR17, O4, MAP1B, and MBP (Fig. 3). Of these, GPR17 is a new oligodendroglial marker expressed in early OPCs up to the stage of immature pre-oligodendrocytes^22^ and now widely adopted to specifically label OPCs at an intermediate differentiation stage partially overlapping with O4 (Fig. 3; refs 23, 35, 36, 37, 38). O4 is instead a well-established marker of immature oligodendrocytes^39^. MAP1B is the major microtubule associated protein found in microtubules early during development^40^. In oligodendrocytes, MAP1B expression is slightly delayed compared to O4 and immediately precedes morphological differentiation, after which MAP1B is strongly down-regulated^41^. Finally, MBP is known to label mature oligodendrocytes, being a protein associated to myelin^42^. When miR-125a-3p was overexpressed by mimic transfection in the presence of T3 (“strong” differentiation protocol), block of terminal maturation was accompanied only by a strong reduction of MAP1B and MBP expression, whereas earlier markers were not affected. When miR-125a-3p overexpression was performed using the “mild” differentiation protocol (i.e., in the absence of T3), OPCs proceeded slower along their lineage and late antigens were not visible, but the number of earlier GPR17^+^ and MAP1B^+^ precursors was also diminished, to suggest that, under these conditions, miR-125a-3p can regulate OPC maturation not only downstream but also upstream to GPR17.

These data show that when miR-125a-3p levels are high, oligodendrocyte maturation is blocked, and this could explain the high expression levels found during embryogenesis, to suggest that miR-125a-3p may, at the same time, promote neural specification and prevent the untimely expression of genes necessary for oligodendrocyte commitment/maturation and the subsequent myelination process that, indeed does not take place at this stage. Globally these findings show that the biological role of miR-125a-3p changes depending on the developmental stage, and that, from embryonic life to adulthood, its expression moves from brain ventricles to parenchyma up to neurons of the cerebral cortex, where it is likely to repress the expression of non-neuronal genes.

Several other transcripts involved in pathways other than myelination have been so far identified as direct targets of miR-125a-3p. By using the Ingenuity Pathway Analysis tool, we have been able to connect several of these players to each other, building a model for the synergic regulation of MBP and myelination (Fig. 4). Among these players, FYN-kinase plays important roles in neuronal functions, myelination, oligodendrocytes formation and cytoskeletal rearrangements^43^. NRG1 is a potent chemoattractant that selectively regulates OPC migration during early CNS development stages of CNS through interaction with the ErbB4 receptor^44^. In line with the prediction of our IPA analysis, overexpression of miR-125a-3p resulted in a reduction of Fyn and Nrg1 and Map1b. It is worth to note that despite a striking effect on MBP expression, the reduction of these genes was quantitatively smaller, supporting the hypothesis that the mechanism of action consists in a fine modulation of a network of genes. Indeed, several reports, in which physical interaction between a miRNA and specific binding sites on target transcripts have been demonstrated by 3′UTR reporter assay, showed a 20% reduction of the target mRNA^45^. It is also worth to note that miRNAs do not necessarily degrade their target mRNAs, but they can also impair their translation into protein^46^, thus, the downregulation observed by qRT-PCR may not fully reflect the observed functional effect.

These data are also consistent with the previous demonstration that the mRNAs of both Map1b and Mbp are stabilized by the RNA-binding protein QKI, which is, in turn, regulated by FYN-Kinase. Lack of FYN-kinase, as detected after mimic transfection, leads to failure of MBP to incorporate into myelin^17^. Moreover, FYN activation is mediated by phosphorylation of a tyrosine residue (Y420), which, in turn, depends on the interaction between NRG1 (which is downregulated after mimic transfection) and its receptor ERBB4^47^. All these data support the hypothesis that miR-125a-3p synergistically inhibits different mechanisms that normally promote the expression of myelin genes. Thus, it is not surprising that miR-125a-3p can strongly impair myelination via the forced inhibition of multiple targets.

The identification of miR-125a-3p as a modulator of oligodendrocyte differentiation provide new findings about the complex regulation of myelination processes and it is conceivable that an antago-miRNA specific for this miRNA may help in promoting oligodendrocyte maturation in diseases characterized by impaired myelin repair.

Reports from oncology, cardiovascular research and infectivology have demonstrated the potential diagnostic and prognostic significance of miRNAs passively leaked or actively released from cells into biological fluids including circulating blood and CSF^29^, either contributing to disease pathogenesis or reflecting response to treatment. Living neurons, but also oligodendrocytes and other CNS cells secrete miRNAs into the extracellular space packaged in exosomes or microvesicles^48^,^49^. Recent evidence support that miRNAs are altered in bodily fluids in Parkinson’s and Alzheimer’s Disease^50^, suggesting that their release does reflect not only activation of immune cells, but also the undergoing neurodegenerative process.

In a previous study, the -5p arm of miR-125a was found to act as a key regulator of brain endothelial integrity and its levels to be increased in MS brain lesions as compared to surrounding normal appearing white matter^51^. Coherently, treatment of MS patients with the anti-VLA4 monoclonal antibody (Tysabri) that inhibits inflammatory cell migration to the brain parenchyma and thus favours endothelial integrity, results in decreased blood levels of miR-125a-5p^52^. However, up to the present study, changes of the −3p arm of this miRNA had not been investigated. Here we show that miR-125a-3p is up-regulated in the CSF of relapsing MS patients compared to control subjects. This alteration was specific for MS, since it was not found in Alzheimer’s disease patients.

This increase could be explained by passive release of miR-125a-3p from neural cells such as neurons or oligodendrocytes undergoing destruction in active MS patients. We cannot obviously exclude that the aberrant presence of this miRNA in CSF could also reflect the inflammation state of the CNS during the relapsing-remitting phase of the disease^53^, since previous reports have demonstrated the role of miR-125a in immune cells activation^54^.

Here we propose a mechanistic role for miR-125a-3p based on data obtained on cultured rodent OPCs. We are aware that human OPCs do not necessarily behave in the same exact way, since they are very heterogeneous and only some subsets are able to myelinate. Some miRNAs thought to be characteristic of human OPCs are also present in mature oligodendrocytes, suggesting that after differentiation, they may acquire different roles, such as regulation of plasticity for myelin maintenance and repair^55^. For example, miR-138, which is known to be more expressed in mature oligodendrocytes compared to OPCs, induces oligodendrocyte differentiation and expression of CNP and MBP, but in contrast, its sustained expression can lead to a reduction in MOG protein, demonstrating that miRNAs may have opposite effects during the transition from OPCs to mature oligodendrocytes^56^. In this respect, our results suggest that also miR-125a-3p could have a Janus-like role in OPC maturation, acting as a repressor in the early phases of the maturation and likely as a positive factor in later phases, repressing genes that could impair terminal maturation.

Heterogeneity of OPCs will have to be taken in account also when transposing our *in vitro* results to human MS subjects showing increased levels of miR-125a-3p in CSF; however, this does not invalidate the biological importance of our findings in MS patients, since a biomarker is defined as a proxy that allows remote and early detection of a biological process (i.e., disease) regardless of its mechanistic role in the condition being diagnosed^57^. In this perspective, our data could serve as a basis for further studies on larger cohort of patients to validate miR-125a-3p as a biomarker for different stages of MS, providing a previously unrecognized venue for medical interventions.

## Methods

### In silico analysis

For the prediction of miRNA target transcripts we used MyMir^58^ (http://www.itb.cnr.it/micro), a system based on integration, filtering and re-ranking of outputs produced by different miRNA databases (i.e. TargetScan, RNAHybrid, miRanda, PITA). STRING database^59^ was used to obtain a list of the Gene Ontology^60^ biological processes (GO BPs) significantly enriched (p value < 0.05) for miR-125a-3p targets. For the analysis, the false discovery rate (FDR) adjusted p-value was used, to reduce the chance of false-positive results. The fold enrichment in miR-125a-3p was calculated using the following formula as previously described^61^:


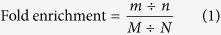


m = genes target in a BP; n = all genes target in the list; M = all genes in a BP; N = all genes in the genome. A fold enrichment greater than 1 (expected value) indicates that the category is overrepresented in the input list. The network model for the synergic regulation of MBP was built with QIAGEN’s Ingenuity^®^ Pathway Analysis (IPA^®^, QIAGEN Redwood City, www.qiagen.com/ingenuity), by inserting both miR-125a-3p predicted and validated targets.

### Primary cultures and OPC isolation

Mixed glial cultures were obtained from 12 postnatal day 2 (P2) Sprague-Dawley rat cerebral cortices pooled together. The shaking protocol allowed the sequential isolation of OPCs, astrocytes and microglia from the same preparation^62^. In these conditions we obtained approximately 600,000 OPCs from each pup; contaminating astrocytes and microglia were routinely less than 2–3% each.

OPCs were plated onto poly-D,L-ornithine-coated (final concentration 50 μg/ml; Sigma-Aldrich) 13-mm glass coverslips for immunocytochemistry (2–3 × 10^4^ cells/coverslip) and poly-D,L-ornithine-coated 6-wells plates (10^5^ cells/coverslip) or 6-cm dishes (2.5 × 10^5^ cells/coverslip) for qRT-PCR assays. Cells were plated in Neurobasal medium supplemented with 2% B27 (Life Technologies), 2 mM L-glutamine, 10 ng/ml human platelet-derived growth factor BB (Sigma-Aldrich), and 10 ng/ml human basic fibroblast growth factor (Life Technologies) to promote proliferation. When OPCs reached a 60% confluency, cultures were switched to a Neurobasal medium lacking growth factors and containing triiodothyronine 15 nM (T3, Sigma-Aldrich) to allow differentiation. In selected experiments, T3 was not added to induce a slower maturation.

The primary cultures used for the experiments were derived from rats. All the procedures were performed in accordance with the approved national law, with the implementation of European Union Directive nr. 86/609/CEE regarding the Protection of animals used for experimental research.

The experiments were approved by the Council of the Dipartimento di Scienze Farmacologiche e Biomolecolari, Università degli Studi di Milano, Italy, which is legally entitled for the use of animals for scientific proposes (D.M. of Italian Ministry of Health, Authorization #295/2012-A 12/20/2012, according to the D.lgs 116/92).

### Transfection of primary OPCs with miR-125a-3p mimics and inhibitors

OPCs were transfected immediately after switching from proliferating to differentiating medium (in the absence or in the presence of T3). MiR-125a-3p mimics or hairpin inhibitors (Dharmacon) were transfected at the final concentration of 50 nM. A scrambled miRNA transfection was included as negative control. MiRNAs were transfected with Lipofectamine RNAiMAX reagent (Life Technologies) following the manufacturer’s protocol.

### Immunocytochemistry and cell counting

Cells were fixed in a 4% paraformaldehyde phosphate-buffered solution containing 4% sucrose. The following primary antibodies were used: rabbit anti-Olig2 (1:100; Millipore), rabbit anti-GPR17 (1:100; Cayman Chemical), rat anti-MBP (1:200; Merck Millipore), goat anti-MAP1B (1:300; Santa Cruz), mouse anti-O4 (1:100; R&D), mouse anti-GFAP (1:100; Cell Signaling). To identify microglial cells, coverlips were incubated with isolectin B4 FITC conjugated (1:100; Sigma). Incubation of primary antibodies were performed 2.5 hours at room temperature or over-night at 4 °C. Cells were then incubated for 1 h at room temperature with secondary antibodies conjugated to either AlexaFluor 488 or AlexaFluor 555 (1:600; Life Technologies). All the antibodies were diluted in a phosphate-buffered blocking solution (pH 7.4) containing 0.3% Triton X-100, with the exception of O4, which did not require detergents. Nuclei were labeled with the UV fluorescent dye Hoechst 33258 (1:10,000; Life Technologies). Coverslips were then mounted in a fluorescent mounting medium (Dako). Olig2^+^ cells were considered as oligodendroglial cells (see Supplementary Figs S2 and S3 for representative micrographs). Positive cells for the selected markers were counted from 20 random fields for each coverslip (0.07 mm^2^/field). The result was expressed as a percentage over the number of nuclei, and then normalized versus controls (transfected with negative miRNA) set to 100%. In the presence of T3, in control conditions, we counted a mean of 880 nuclei, 124 GPR17^+^, 330 O4^+^, 282 MAP1B^+^ and 54 MBP^+^ cells for each coverslip. In the absence of T3, in control conditions, we counted a mean of 848 nuclei, 138 GPR17^+^ and 217 MAP1B^+^ cells for each coverslip.

### MicroRNA *in situ* hybridization (ISH) in combination with immunohistochemistry

After anesthesia, post-natal P7, P14 and adult rats were perfused with 4% paraformaldehyde (PFA; Sigma) in phosphate buffered saline (PBS). Brains were explanted and post-fixed in 4% PFA for 1 hour. Embryos and P2 rat brains were fixed by immersion in 4% PFA for 4 hours. Tissues were washed in PBS, dehydrated overnight in 30% sucrose, and then embedded in OCT (Bio-optica). Twelve micrometer sections were cut on a cryostat in RNase-free conditions, transferred to Superfrost/plus microscope slides (Thermo Scientific), and stored at 20 °C. Buffers and reagents were prepared using DEPC-treated (Sigma) water and autoclaved. Detection of miRNA was performed with miRCURY LNA miRNA detection probes (Exiqon), using a previously described protocol^63^ with minor modifications. Briefly, tissues were further fixed in 10% formalin (Sigma) and then subjected to acetylation. Pre-hybridization was carried out for 2 hours at room temperature, followed by hybridization with scramble/miR-125a-3p DIG-LNA probes (20 nM; Exiqon) overnight at 55 °C. The following day, endogenous peroxidases were blocked with 1% H_2_O_2_ (Sigma). For probe detection, the sections were incubated with a sheep anti-DIG-POD primary antibody (1:600; Roche), diluted in blocking buffer (0.05% Tween-20, 1% BSA and 1% sheep serum, all from Sigma) at room temperature for 2 hours, then with biotynilated-tyramide for signal amplification (Perkin Elmer) and finally streptavidin Alexa Fluor 555 conjugated (1:1000; Life Technologies) for 50 minutes.

For detecting the epitopes of interest, immunohistochemistry (IHC) was combined with ISH by using the following primary antibodies: rabbit anti-Olig2 (1:100; Millipore), rabbit anti-Iba1 (1:500; Wako), mouse anti-GFAP (1:100; Cell Signaling), mouse anti-NeuN (1:100; Millipore), mouse anti-Nestin (1:100; Millipore), rabbit anti-NG2 (1:100; Millipore), mouse anti-CC1 (1:50; Calbiochem). Alexa Fluor 488 or 633 (1:600 and 1:300, respectively; Life Technologies), were used as secondary antibodies in double or triple staining. Stainings with Alexa Fluor 633 were always shown in either white or blue pseudocolor. Nuclei were labeled with the UV fluorescent dye Hoechst 33258 (1:20,000; Life Technologies). Finally, slides were mounted in a fluorescent mounting medium (Dako).

### Total RNA extraction, retrotranscription and gene expression analysis

Total RNA was extracted from cells or tissues using Trizol reagent (Life Technologies). RNA from neural cells was harvested from *in vitro* cultures (see above). RNAs from human tissues were purchased from Ambion. For qRT-PCR of miRNA, 10 ng of total RNA was reverse-transcribed with TaqMan^®^ MicroRNA Reverse Transcription Kit and then subjected to Taqman microRNA assay (Life Technologies). Expression level of miR-125a-3p was normalized to the U87 snRNA in rat and to the U6 snRNA in human by the ΔCt method. For gene expression analysis, cDNA synthesis was performed starting from 800 ng of total RNA using SuperScript^®^ II Reverse Transcriptase (Life Technologies). The expression of all genes was analyzed with TaqMan^®^ Gene Expression Assays and normalized to GAPDH expression using CFX96 real time PCR system (Bio-rad) following the manufacturer’s protocol.

### Isolation of miRNAs from cerebrospinal fluid (CSF) and real-time PCR quantification assays

Human samples of CSF have been obtained from the Institute of Experimental Neurology (INSpe) Biobank at the San Raffaele Hospital after thorough evaluation of neuropathology and consisted of MS, Alzheimer, and neurologically normal control subjects. The distinction between patients who had radiologic or clinical evidence of MS activity was done through the assessment of gadolinium-enhancing lesions on cranial MRI and presence of recent clinical relapses^64^. According to the recently updated McDonald criteria^65^, patients have been considered to be in a clinical relapsing phase of the disease if they experienced a relapse in the 30 days prior to lumbar puncture. Patients with one or more gadolinium-enhancing lesions at baseline MRI (with or without corresponding clinical symptoms) were classified the same as in a relapsing (active) phase of the disease.

The specimens were collected from adult subjects under informed consent in accordance with the laws and with the internal guidelines and regulations of the San Raffaele Hospital. The protocols were approved by the Ethical commitee of the San Raffaele Hospital. The analysis on human specimens were also approved by the Ethical Committee of the University of Milan (prot. 71/14). CSF samples were collected by diagnostic lumbar puncture or puncture of Ommaya reservoirs from individual MS patients, centrifuged at 13,000 g for 5 minutes and stored at −80 °C. RNA was isolated from 200 μl of CSF using miRNeasy Serum/Plasma Kit (Qiagen, Milan) following the manufacturer’s protocol and reverse-transcribed with TaqMan^®^ MicroRNA Reverse Transcription Kit (Life Technologies). Expression level of miR-125a-3p was analyzed with TaqMan^®^ MicroRNA Assays and normalized to spiked-in cel-miR-39 by the ΔCt method.

### Statistical analysis

Data are presented as mean ± standard error (SEM) of replicates. GraphPad Prism 6.0 was used for statistical analysis. For all comparisons between two groups with a normal distribution, two-tailed unpaired t-test was performed. For multiple comparison testing, one-way analysis of variance (ANOVA) accompanied by Dunnett’s post-hoc test were used. For CSF samples, data with a Median Absolute Deviation (MAD) >3.5 were considered outliers and discarded from the analysis. P values < 0.05 were considered statistically significant.

## Additional Information

**How to cite this article**: Lecca, D. *et al*. MiR-125a-3p timely inhibits oligodendroglial maturation and is pathologically up-regulated in human multiple sclerosis. *Sci. Rep.* **6**, 34503; doi: 10.1038/srep34503 (2016).

## Supplementary Material

Supplementary Information

## Figures and Tables

**Table 1:** Demographic data and miR-125a-3p log2 fold increase by diagnostic group.

Diagnostic group	n	Sex F:M	Age y, mean	miR-125a-3p log2 fold increase	p-value
CTRL	13	1.6	75.9	0.000 ± 0.141	—
AD	17	2.4	72.6	0.030 ± 0.125	0.731
Active-MS	11	1.75	32.1	0.700 ± 0.185	0.004
Inactive-MS	19	3.75	34.8	0.152 ± 0.178	0.452

P-values were obtained applying unpaired t-test compared to control group.
