# Supplementary material for: MiR-125a-3p timely inhibits oligodendroglial maturation and is pathologically up-regulated in human multiple sclerosis
Source: Sci Rep. 2016 Oct 4;6:34503. doi: 10.1038/srep34503 (PMC5048305; doi:10.1038/srep34503)
Supplement: Supplementary Information [file srep34503-s1.pdf]

# MiR-125a-3p timely inhibits oligodendroglial maturation and is pathologically up-regulated in human multiple sclerosis

Davide Lecca[1¶], Davide Marangon[1¶], Giusy T. Coppolino[1], Aida Menéndez Méndez[3], Annamaria Finardi[2], Gloria Dalla Costa[2], Vittorio Martinelli[2], Roberto Furlan[2], Maria P. Abbracchio[1*].

[1] Laboratory of Molecular and Cellular Pharmacology of the Purinergic Transmission, Dipartimento di Scienze Farmacologiche e Biomolecolari, Università degli Studi di Milano, Milan, 20133, Italy.

[2] Institute of Experimental Neurology (INSpe), Division of Neuroscience, San Raffaele Scientific Institute, Milan, 20132, Italy.

[3] Departamento de Bioquímica y Biología Molecular IV, Universidad Complutense de Madrid, 28040, Spain.

[¶] These authors equally contributed

**Supplementary Table S1.** List of all Gene Ontology biological processes enriched in miR-125a-3p targets.

| GO_id | Term | N of genes | p-value (fdr) |
|---|---|---|---|
| GO:0009987 | cellular process | 820 | 5.25E-31 |
| GO:0044763 | single-organism cellular process | 706 | 5.6E-31 |
| GO:0065007 | biological regulation | 664 | 4,00E-25 |
| GO:0008150 | biological_process | 847 | 9.16E-25 |
| GO:0044699 | single-organism process | 731 | 2.07E-24 |
| GO:0050789 | regulation of biological process | 638 | 3.82E-24 |
| GO:0050794 | regulation of cellular process | 601 | 5.22E-21 |
| GO:0008152 | metabolic process | 590 | 6.29E-15 |
| GO:0048731 | system development | 307 | 3.54E-14 |
| GO:0007399 | nervous system development | 185 | 1.51E-13 |
| GO:0044237 | cellular metabolic process | 510 | 2.96E-13 |
| GO:0007275 | multicellular organismal development | 336 | 7.08E-13 |
| GO:0051716 | cellular response to stimulus | 359 | 1.65E-12 |
| GO:0044767 | single-organism developmental process | 369 | 3.43E-12 |
| GO:0071704 | organic substance metabolic process | 527 | 4.87E-12 |
| GO:0044700 | single organism signaling | 294 | 8.09E-12 |
| GO:0032502 | developmental process | 368 | 8.45E-12 |
| GO:0048519 | negative regulation of biological process | 321 | 1.32E-11 |
| GO:0007154 | cell communication | 301 | 1.37E-11 |
| GO:0044238 | primary metabolic process | 510 | 2.93E-11 |
| GO:0048518 | positive regulation of biological process | 368 | 3.13E-11 |

| | | | |
|---|---|---|---|
| GO:0019222 | regulation of metabolic process | 416 | 3.23E-11 |
| GO:0048523 | negative regulation of cellular process | 300 | 3.49E-11 |
| GO:0016043 | cellular component organization | 318 | 8.12E-11 |
| GO:0048513 | organ development | 238 | 9.98E-11 |
| GO:0043170 | macromolecule metabolic process | 441 | 1.15E-10 |
| GO:0048856 | anatomical structure development | 330 | 1.69E-10 |
| GO:0044707 | single-multicellular organism process | 380 | 2.08E-10 |
| GO:0010646 | regulation of cell communication | 218 | 2.55E-10 |
| GO:0048522 | positive regulation of cellular process | 325 | 2.81E-10 |
| GO:0071840 | cellular component organization or biogenesis | 324 | 3.18E-10 |
| GO:0044260 | cellular macromolecule metabolic process | 408 | 3.93E-10 |
| GO:0048583 | regulation of response to stimulus | 238 | 5.37E-10 |
| GO:0007165 | signal transduction | 271 | 6,00E-10 |
| GO:0050896 | response to stimulus | 407 | 6,00E-10 |
| GO:0051179 | localization | 309 | 7.13E-10 |
| GO:0032501 | multicellular organismal process | 386 | 9.91E-10 |
| GO:0023051 | regulation of signaling | 206 | 1.38E-9 |
| GO:0051128 | regulation of cellular component organization | 184 | 1.67E-9 |
| GO:0022008 | neurogenesis | 132 | 2.26E-9 |
| GO:0009966 | regulation of signal transduction | 184 | 5.94E-9 |
| GO:0031323 | regulation of cellular metabolic process | 361 | 1.14E-8 |
| GO:0048699 | generation of neurons | 123 | 1.14E-8 |
| GO:0006464 | cellular protein modification process | 197 | 2.1E-8 |
| GO:0043412 | macromolecule modification | 205 | 2.24E-8 |
| GO:0032879 | regulation of localization | 182 | 2.61E-8 |
| GO:0030154 | cell differentiation | 250 | 4.17E-8 |
| GO:0044267 | cellular protein metabolic process | 233 | 4.17E-8 |
| GO:0080090 | regulation of primary metabolic process | 348 | 4.69E-8 |
| GO:0019538 | protein metabolic process | 267 | 4.83E-8 |
| GO:0007166 | cell surface receptor signaling pathway | 135 | 7.6E-8 |
| GO:0035556 | intracellular signal transduction | 123 | 8.61E-8 |
| GO:0043067 | regulation of programmed cell death | 122 | 8.77E-8 |
| GO:0000904 | cell morphogenesis involved in differentiation | 62 | 9.35E-8 |
| GO:0010941 | regulation of cell death | 127 | 1.24E-7 |
| GO:0065008 | regulation of biological quality | 209 | 1.69E-7 |
| GO:0060255 | regulation of macromolecule metabolic process | 344 | 1.7E-7 |
| GO:1902578 | single-organism localization | 209 | 1.82E-7 |
| GO:0048869 | cellular developmental process | 257 | 3.03E-7 |
| GO:0044765 | single-organism transport | 195 | 3.91E-7 |
| GO:0051234 | establishment of localization | 243 | 4.94E-7 |
| GO:0042981 | regulation of apoptotic process | 118 | 5.21E-7 |
| GO:0030182 | neuron differentiation | 88 | 5.21E-7 |
| GO:0006810 | transport | 235 | 5.74E-7 |
| GO:0009653 | anatomical structure morphogenesis | 174 | 1,00E-06 |
| GO:0006996 | organelle organization | 201 | 1.01E-6 |
| GO:0009968 | negative regulation of signal transduction | 93 | 1.22E-6 |

| | | | |
|---|---|---|---|
| GO:0023057 | negative regulation of signaling | 101 | 1.62E-6 |
| GO:0048468 | cell development | 135 | 1.78E-6 |
| GO:0044710 | single-organism metabolic process | 257 | 1.78E-6 |
| GO:0010648 | negative regulation of cell communication | 101 | 2.27E-6 |
| GO:0009893 | positive regulation of metabolic process | 245 | 2.56E-6 |
| GO:0065009 | regulation of molecular function | 189 | 2.8E-6 |
| GO:0012501 | programmed cell death | 89 | 3.25E-6 |
| GO:0031175 | neuron projection development | 59 | 3.74E-6 |
| GO:0006793 | phosphorus metabolic process | 143 | 3.78E-6 |
| GO:0007417 | central nervous system development | 85 | 3.8E-6 |
| GO:0051049 | regulation of transport | 136 | 4.01E-6 |
| GO:0006915 | apoptotic process | 87 | 4.01E-6 |
| GO:0048666 | neuron development | 71 | 5.06E-6 |
| GO:2000811 | negative regulation of anoikis | 9 | 5.33E-6 |
| GO:0048667 | cell morphogenesis involved in neuron differentiation | 44 | 6.06E-6 |
| GO:0008219 | cell death | 90 | 6.89E-6 |
| GO:0016265 | death | 90 | 6.89E-6 |
| GO:0051246 | regulation of protein metabolic process | 183 | 7.42E-6 |
| GO:0048812 | neuron projection morphogenesis | 46 | 8.35E-6 |
| GO:0048585 | negative regulation of response to stimulus | 108 | 9.01E-6 |
| GO:0016310 | phosphorylation | 95 | 1.05E-5 |
| GO:0009892 | negative regulation of metabolic process | 182 | 1.26E-5 |
| GO:0051239 | regulation of multicellular organismal process | 183 | 1.52E-5 |
| GO:0051640 | organelle localization | 43 | 1.52E-5 |
| GO:0007409 | axonogenesis | 38 | 1.52E-5 |
| GO:0010604 | positive regulation of macromolecule metabolic process | 195 | 1.57E-5 |
| GO:1902531 | regulation of intracellular signal transduction | 110 | 1.67E-5 |
| GO:0043069 | negative regulation of programmed cell death | 78 | 1.71E-5 |
| GO:0006796 | phosphate-containing compound metabolic process | 137 | 1.79E-5 |
| GO:0031325 | positive regulation of cellular metabolic process | 204 | 2.3E-5 |
| GO:0010647 | positive regulation of cell communication | 123 | 2.48E-5 |
| GO:0051641 | cellular localization | 143 | 2.63E-5 |
| GO:0032268 | regulation of cellular protein metabolic process | 169 | 2.66E-5 |
| GO:0006468 | protein phosphorylation | 78 | 2.86E-5 |
| GO:0061564 | axon development | 39 | 3.64E-5 |
| GO:0060548 | negative regulation of cell death | 81 | 3.79E-5 |
| GO:0023056 | positive regulation of signaling | 114 | 4.14E-5 |
| GO:0050790 | regulation of catalytic activity | 153 | 4.32E-5 |
| GO:0042127 | regulation of cell proliferation | 116 | 5.49E-5 |
| GO:0007420 | brain development | 68 | 5.84E-5 |
| GO:0060322 | head development | 71 | 5.85E-5 |
| GO:2000209 | regulation of anoikis | 9 | 5.92E-5 |
| GO:0031399 | regulation of protein modification process | 123 | 6.31E-5 |
| GO:0043933 | macromolecular complex subunit organization | 137 | 8.15E-5 |
| GO:0033554 | cellular response to stress | 115 | 9,00E-05 |
| GO:0060341 | regulation of cellular localization | 97 | 1.02E-4 |

| | | | |
|---|---|---|---|
| GO:0071702 | organic substance transport | 127 | 1.02E-4 |
| GO:0043066 | negative regulation of apoptotic process | 74 | 1.11E-4 |
| GO:0010001 | glial cell differentiation | 24 | 1.12E-4 |
| GO:0010605 | negative regulation of macromolecule metabolic process | 162 | 1.29E-4 |
| GO:0009888 | tissue development | 133 | 1.29E-4 |
| GO:0048584 | positive regulation of response to stimulus | 133 | 1.53E-4 |
| GO:0051656 | establishment of organelle localization | 34 | 1.56E-4 |
| GO:0031401 | positive regulation of protein modification process | 84 | 1.63E-4 |
| GO:0045595 | regulation of cell differentiation | 122 | 1.69E-4 |
| GO:0006950 | response to stress | 195 | 1.69E-4 |
| GO:0044093 | positive regulation of molecular function | 119 | 1.69E-4 |
| GO:0001775 | cell activation | 54 | 1.86E-4 |
| GO:0051270 | regulation of cellular component movement | 66 | 1.94E-4 |
| GO:0071822 | protein complex subunit organization | 95 | 1.94E-4 |
| GO:0040011 | locomotion | 88 | 1.94E-4 |
| GO:0009967 | positive regulation of signal transduction | 101 | 1.94E-4 |
| GO:0051130 | positive regulation of cellular component organization | 97 | 1.95E-4 |
| GO:0060284 | regulation of cell development | 74 | 1.95E-4 |
| GO:0032989 | cellular component morphogenesis | 82 | 2.08E-4 |
| GO:0006928 | movement of cell or subcellular component | 93 | 2.08E-4 |
| GO:0031324 | negative regulation of cellular metabolic process | 161 | 2.14E-4 |
| GO:0044723 | single-organism carbohydrate metabolic process | 52 | 2.36E-4 |
| GO:0042063 | gliogenesis | 27 | 2.46E-4 |
| GO:0010033 | response to organic substance | 153 | 2.76E-4 |
| GO:0051247 | positive regulation of protein metabolic process | 106 | 2.8E-4 |
| GO:0051726 | regulation of cell cycle | 77 | 2.84E-4 |
| GO:0050793 | regulation of developmental process | 161 | 2.86E-4 |
| GO:0044802 | single-organism membrane organization | 54 | 2.95E-4 |
| GO:0016192 | vesicle-mediated transport | 84 | 3.09E-4 |
| GO:0008284 | positive regulation of cell proliferation | 71 | 3.16E-4 |
| GO:0044249 | cellular biosynthetic process | 252 | 3.16E-4 |
| GO:0032270 | positive regulation of cellular protein metabolic process | 99 | 3.74E-4 |
| GO:0001934 | positive regulation of protein phosphorylation | 70 | 4.38E-4 |
| GO:0034645 | cellular macromolecule biosynthetic process | 208 | 4.38E-4 |
| GO:0051050 | positive regulation of transport | 75 | 4.51E-4 |
| GO:0007264 | small GTPase mediated signal transduction | 38 | 4.64E-4 |
| GO:0000902 | cell morphogenesis | 74 | 4.65E-4 |
| GO:1901576 | organic substance biosynthetic process | 255 | 4.86E-4 |
| GO:0010721 | negative regulation of cell development | 34 | 5,00E-04 |
| GO:0022610 | biological adhesion | 81 | 5.02E-4 |
| GO:0042327 | positive regulation of phosphorylation | 72 | 5.18E-4 |
| GO:0070828 | heterochromatin organization | 7 | 5.29E-4 |
| GO:0007167 | enzyme linked receptor protein signaling pathway | 53 | 5.33E-4 |
| GO:0007169 | transmembrane receptor protein tyrosine kinase signaling pathway | 39 | 5.48E-4 |
| GO:0016477 | cell migration | 65 | 5.65E-4 |
| GO:0006355 | regulation of transcription, DNA-templated | 205 | 5.7E-4 |

| | | | |
|---|---|---|---|
| GO:0009059 | macromolecule biosynthetic process | 208 | 6.34E-4 |
| GO:0009058 | biosynthetic process | 259 | 6.57E-4 |
| GO:0050767 | regulation of neurogenesis | 60 | 6.77E-4 |
| GO:0007155 | cell adhesion | 80 | 6.8E-4 |
| GO:0009889 | regulation of biosynthetic process | 241 | 7.13E-4 |
| GO:0009894 | regulation of catabolic process | 65 | 7.78E-4 |
| GO:0046907 | intracellular transport | 88 | 8.24E-4 |
| GO:1903506 | regulation of nucleic acid-templated transcription | 204 | 8.68E-4 |
| GO:0001944 | vasculature development | 51 | 8.72E-4 |
| GO:0005975 | carbohydrate metabolic process | 56 | 8.96E-4 |
| GO:0022607 | cellular component assembly | 123 | 9.15E-4 |
| GO:0019438 | aromatic compound biosynthetic process | 176 | 9.36E-4 |
| GO:0040012 | regulation of locomotion | 63 | 9.36E-4 |
| GO:0031326 | regulation of cellular biosynthetic process | 237 | 9.8E-4 |
| GO:0033043 | regulation of organelle organization | 89 | 9.83E-4 |
| GO:2001141 | regulation of RNA biosynthetic process | 204 | 9.83E-4 |
| GO:1901698 | response to nitrogen compound | 67 | 1.01E-3 |
| GO:2000112 | regulation of cellular macromolecule biosynthetic process | 221 | 1.07E-3 |
| GO:0042221 | response to chemical | 193 | 1.07E-3 |
| GO:0018130 | heterocycle biosynthetic process | 175 | 1.07E-3 |
| GO:0051649 | establishment of localization in cell | 110 | 1.15E-3 |
| GO:2000145 | regulation of cell motility | 58 | 1.16E-3 |
| GO:0030334 | regulation of cell migration | 56 | 1.16E-3 |
| GO:0030030 | cell projection organization | 76 | 1.16E-3 |
| GO:0045664 | regulation of neuron differentiation | 50 | 1.16E-3 |
| GO:0061024 | membrane organization | 57 | 1.17E-3 |
| GO:0050768 | negative regulation of neurogenesis | 28 | 1.17E-3 |
| GO:0051674 | localization of cell | 69 | 1.21E-3 |
| GO:1902533 | positive regulation of intracellular signal transduction | 70 | 1.21E-3 |
| GO:0045321 | leukocyte activation | 44 | 1.23E-3 |
| GO:0048870 | cell motility | 69 | 1.25E-3 |
| GO:0010468 | regulation of gene expression | 238 | 1.28E-3 |
| GO:0072358 | cardiovascular system development | 73 | 1.28E-3 |
| GO:0072359 | circulatory system development | 73 | 1.28E-3 |
| GO:0051302 | regulation of cell division | 31 | 1.3E-3 |
| GO:0008366 | axon ensheathment | 16 | 1.3E-3 |
| GO:0030900 | forebrain development | 41 | 1.3E-3 |
| GO:0034654 | nucleobase-containing compound biosynthetic process | 170 | 1.3E-3 |
| GO:0035295 | tube development | 61 | 1.31E-3 |
| GO:0009719 | response to endogenous stimulus | 96 | 1.32E-3 |
| GO:0051047 | positive regulation of secretion | 37 | 1.32E-3 |
| GO:0043408 | regulation of MAPK cascade | 56 | 1.38E-3 |
| GO:0051650 | establishment of vesicle localization | 22 | 1.42E-3 |
| GO:0050853 | B cell receptor signaling pathway | 9 | 1.46E-3 |
| GO:1902589 | single-organism organelle organization | 138 | 1.52E-3 |
| GO:0051252 | regulation of RNA metabolic process | 208 | 1.53E-3 |

| | | | |
|---|---|---|---|
| GO:0009056 | catabolic process | 108 | 1.55E-3 |
| GO:0010556 | regulation of macromolecule biosynthetic process | 225 | 1.57E-3 |
| GO:0045596 | negative regulation of cell differentiation | 60 | 1.67E-3 |
| GO:1902580 | single-organism cellular localization | 61 | 1.67E-3 |
| GO:0006351 | transcription, DNA-templated | 153 | 1.67E-3 |
| GO:0032402 | melanosome transport | 7 | 1.87E-3 |
| GO:0044248 | cellular catabolic process | 93 | 1.87E-3 |
| GO:0022603 | regulation of anatomical structure morphogenesis | 75 | 2.08E-3 |
| GO:0070085 | glycosylation | 28 | 2.12E-3 |
| GO:0060429 | epithelium development | 86 | 2.17E-3 |
| GO:0033036 | macromolecule localization | 132 | 2.22E-3 |
| GO:0032774 | RNA biosynthetic process | 153 | 2.3E-3 |
| GO:1903320 | regulation of protein modification by small protein conjugation or removal | 26 | 2.39E-3 |
| GO:0016050 | vesicle organization | 24 | 2.4E-3 |
| GO:0046777 | protein autophosphorylation | 25 | 2.51E-3 |
| GO:0007010 | cytoskeleton organization | 70 | 2.52E-3 |
| GO:0001568 | blood vessel development | 47 | 2.55E-3 |
| GO:0001655 | urogenital system development | 36 | 2.55E-3 |
| GO:0007265 | Ras protein signal transduction | 24 | 2.6E-3 |
| GO:2001236 | regulation of extrinsic apoptotic signaling pathway | 23 | 2.68E-3 |
| GO:0001932 | regulation of protein phosphorylation | 92 | 2.68E-3 |
| GO:1901362 | organic cyclic compound biosynthetic process | 179 | 2.68E-3 |
| GO:0032401 | establishment of melanosome localization | 7 | 2.68E-3 |
| GO:0051904 | pigment granule transport | 7 | 2.68E-3 |
| GO:0046579 | positive regulation of Ras protein signal transduction | 8 | 2.75E-3 |
| GO:0051098 | regulation of binding | 30 | 2.75E-3 |
| GO:0006486 | protein glycosylation | 27 | 2.75E-3 |
| GO:0043413 | macromolecule glycosylation | 27 | 2.75E-3 |
| GO:0031396 | regulation of protein ubiquitination | 24 | 2.75E-3 |
| GO:0030097 | hemopoiesis | 54 | 2.76E-3 |
| GO:0080134 | regulation of response to stress | 89 | 2.78E-3 |
| GO:0010564 | regulation of cell cycle process | 45 | 2.78E-3 |
| GO:0051171 | regulation of nitrogen compound metabolic process | 236 | 2.78E-3 |
| GO:0032880 | regulation of protein localization | 73 | 2.79E-3 |
| GO:0070372 | regulation of ERK1 and ERK2 cascade | 25 | 2.79E-3 |
| GO:0051046 | regulation of secretion | 57 | 2.8E-3 |
| GO:0018193 | peptidyl-amino acid modification | 59 | 2.97E-3 |
| GO:0051960 | regulation of nervous system development | 64 | 2.97E-3 |
| GO:0042552 | myelination | 15 | 3.03E-3 |
| GO:0006357 | regulation of transcription from RNA polymerase II promoter | 126 | 3.03E-3 |
| GO:0030335 | positive regulation of cell migration | 36 | 3.06E-3 |
| GO:0043085 | positive regulation of catalytic activity | 95 | 3.1E-3 |
| GO:0031344 | regulation of cell projection organization | 46 | 3.1E-3 |
| GO:0051648 | vesicle localization | 22 | 3.1E-3 |
| GO:0010243 | response to organonitrogen compound | 59 | 3.17E-3 |
| GO:0001763 | morphogenesis of a branching structure | 26 | 3.29E-3 |

| | | | |
|---|---|---|---|
| GO:1902532 | negative regulation of intracellular signal transduction | 40 | 3.29E-3 |
| GO:0045665 | negative regulation of neuron differentiation | 22 | 3.39E-3 |
| GO:0098609 | cell-cell adhesion | 50 | 3.42E-3 |
| GO:0042592 | homeostatic process | 97 | 3.47E-3 |
| GO:0070271 | protein complex biogenesis | 70 | 3.47E-3 |
| GO:0006461 | protein complex assembly | 70 | 3.47E-3 |
| GO:2000026 | regulation of multicellular organismal development | 120 | 3.52E-3 |
| GO:0051905 | establishment of pigment granule localization | 7 | 3.56E-3 |
| GO:0060562 | epithelial tube morphogenesis | 37 | 3.59E-3 |
| GO:1901700 | response to oxygen-containing compound | 93 | 3.59E-3 |
| GO:0042325 | regulation of phosphorylation | 97 | 3.64E-3 |
| GO:0045893 | positive regulation of transcription, DNA-templated | 110 | 3.75E-3 |
| GO:1903508 | positive regulation of nucleic acid-templated transcription | 110 | 3.75E-3 |
| GO:0051272 | positive regulation of cellular component movement | 37 | 3.75E-3 |
| GO:0007423 | sensory organ development | 52 | 3.75E-3 |
| GO:0043410 | positive regulation of MAPK cascade | 40 | 3.75E-3 |
| GO:1902582 | single-organism intracellular transport | 73 | 3.76E-3 |
| GO:0006488 | dolichol-linked oligosaccharide biosynthetic process | 5 | 3.78E-3 |
| GO:0022604 | regulation of cell morphogenesis | 45 | 3.89E-3 |
| GO:1902680 | positive regulation of RNA biosynthetic process | 110 | 4.05E-3 |
| GO:0051241 | negative regulation of multicellular organismal process | 79 | 4.16E-3 |
| GO:1902591 | single-organism membrane budding | 6 | 4.24E-3 |
| GO:1903426 | regulation of reactive oxygen species biosynthetic process | 12 | 4.36E-3 |
| GO:0035239 | tube morphogenesis | 40 | 4.36E-3 |
| GO:0045937 | positive regulation of phosphate metabolic process | 76 | 4.36E-3 |
| GO:0006090 | pyruvate metabolic process | 11 | 4.45E-3 |
| GO:0048858 | cell projection morphogenesis | 53 | 4.45E-3 |
| GO:0010557 | positive regulation of macromolecule biosynthetic process | 120 | 4.48E-3 |
| GO:0051961 | negative regulation of nervous system development | 28 | 4.48E-3 |
| GO:2000147 | positive regulation of cell motility | 36 | 4.58E-3 |
| GO:0072001 | renal system development | 32 | 4.58E-3 |
| GO:0031329 | regulation of cellular catabolic process | 54 | 4.63E-3 |
| GO:0070374 | positive regulation of ERK1 and ERK2 cascade | 19 | 4.95E-3 |
| GO:1901215 | negative regulation of neuron death | 22 | 4.96E-3 |
| GO:0044085 | cellular component biogenesis | 129 | 4.96E-3 |
| GO:0070887 | cellular response to chemical stimulus | 125 | 5.04E-3 |
| GO:0045859 | regulation of protein kinase activity | 56 | 5.04E-3 |
| GO:0051336 | regulation of hydrolase activity | 88 | 5.13E-3 |
| GO:0051783 | regulation of nuclear division | 20 | 5.31E-3 |
| GO:0010975 | regulation of neuron projection development | 36 | 5.39E-3 |
| GO:0019219 | regulation of nucleobase-containing compound metabolic process | 219 | 5.57E-3 |
| GO:0072384 | organelle transport along microtubule | 8 | 5.58E-3 |
| GO:1901214 | regulation of neuron death | 28 | 5.87E-3 |
| GO:0051099 | positive regulation of binding | 17 | 5.89E-3 |
| GO:0035725 | sodium ion transmembrane transport | 17 | 5.89E-3 |
| GO:0006333 | chromatin assembly or disassembly | 16 | 6.4E-3 |

| | | | |
|---|---|---|---|
| GO:0032400 | melanosome localization | 7 | 6.4E-3 |
| GO:0051668 | localization within membrane | 7 | 6.4E-3 |
| GO:0048534 | hematopoietic or lymphoid organ development | 56 | 6.4E-3 |
| GO:0051248 | negative regulation of protein metabolic process | 77 | 6.4E-3 |
| GO:0002682 | regulation of immune system process | 83 | 6.75E-3 |
| GO:0098602 | single organism cell adhesion | 44 | 6.89E-3 |
| GO:0009101 | glycoprotein biosynthetic process | 29 | 6.91E-3 |
| GO:0032787 | monocarboxylic acid metabolic process | 41 | 7,00E-03 |
| GO:0071310 | cellular response to organic substance | 103 | 7.08E-3 |
| GO:0007346 | regulation of mitotic cell cycle | 40 | 7.1E-3 |
| GO:0080135 | regulation of cellular response to stress | 57 | 7.12E-3 |
| GO:0010769 | regulation of cell morphogenesis involved in differentiation | 31 | 7.2E-3 |
| GO:0065003 | macromolecular complex assembly | 81 | 7.21E-3 |
| GO:0047497 | mitochondrion transport along microtubule | 4 | 7.25E-3 |
| GO:0051173 | positive regulation of nitrogen compound metabolic process | 127 | 7.39E-3 |
| GO:0051254 | positive regulation of RNA metabolic process | 111 | 7.53E-3 |
| GO:1903532 | positive regulation of secretion by cell | 32 | 7.7E-3 |
| GO:0006508 | proteolysis | 78 | 7.96E-3 |
| GO:0061138 | morphogenesis of a branching epithelium | 24 | 8.04E-3 |
| GO:0016568 | chromatin modification | 45 | 8.29E-3 |
| GO:0032990 | cell part morphogenesis | 54 | 8.33E-3 |
| GO:0051875 | pigment granule localization | 7 | 8.34E-3 |
| GO:0045428 | regulation of nitric oxide biosynthetic process | 10 | 8.58E-3 |
| GO:0051057 | positive regulation of small GTPase mediated signal transduction | 8 | 8.81E-3 |
| GO:0090342 | regulation of cell aging | 8 | 8.81E-3 |
| GO:0010629 | negative regulation of gene expression | 105 | 9.17E-3 |
| GO:0010628 | positive regulation of gene expression | 123 | 9.32E-3 |
| GO:1903530 | regulation of secretion by cell | 51 | 9.84E-3 |
| GO:0018108 | peptidyl-tyrosine phosphorylation | 21 | 9.9E-3 |
| GO:0043523 | regulation of neuron apoptotic process | 24 | 9.99E-3 |
| GO:0040017 | positive regulation of locomotion | 36 | 1.01E-2 |
| GO:0018210 | peptidyl-threonine modification | 10 | 1.02E-2 |
| GO:0051240 | positive regulation of multicellular organismal process | 102 | 1.03E-2 |
| GO:0050679 | positive regulation of epithelial cell proliferation | 20 | 1.04E-2 |
| GO:0048754 | branching morphogenesis of an epithelial tube | 21 | 1.07E-2 |
| GO:0009100 | glycoprotein metabolic process | 31 | 1.07E-2 |
| GO:0030029 | actin filament-based process | 38 | 1.07E-2 |
| GO:0009628 | response to abiotic stimulus | 75 | 1.08E-2 |
| GO:0044271 | cellular nitrogen compound biosynthetic process | 189 | 1.09E-2 |
| GO:0030155 | regulation of cell adhesion | 50 | 1.09E-2 |
| GO:0051338 | regulation of transferase activity | 62 | 1.11E-2 |
| GO:1901575 | organic substance catabolic process | 90 | 1.11E-2 |
| GO:0010256 | endomembrane system organization | 37 | 1.14E-2 |
| GO:0018212 | peptidyl-tyrosine modification | 21 | 1.14E-2 |
| GO:0016337 | single organismal cell-cell adhesion | 41 | 1.14E-2 |
| GO:0043524 | negative regulation of neuron apoptotic process | 19 | 1.17E-2 |

| | | | |
|---|---|---|---|
| GO:0072012 | glomerulus vasculature development | 6 | 1.18E-2 |
| GO:0060612 | adipose tissue development | 9 | 1.18E-2 |
| GO:0014070 | response to organic cyclic compound | 65 | 1.24E-2 |
| GO:0001525 | angiogenesis | 32 | 1.26E-2 |
| GO:0030036 | actin cytoskeleton organization | 36 | 1.27E-2 |
| GO:2001233 | regulation of apoptotic signaling pathway | 38 | 1.36E-2 |
| GO:0006814 | sodium ion transport | 20 | 1.43E-2 |
| GO:0030163 | protein catabolic process | 44 | 1.43E-2 |
| GO:0032413 | negative regulation of ion transmembrane transporter activity | 9 | 1.43E-2 |
| GO:0038083 | peptidyl-tyrosine autophosphorylation | 9 | 1.43E-2 |
| GO:0051259 | protein oligomerization | 37 | 1.45E-2 |
| GO:0030888 | regulation of B cell proliferation | 11 | 1.48E-2 |
| GO:0001822 | kidney development | 29 | 1.48E-2 |
| GO:0060537 | muscle tissue development | 32 | 1.48E-2 |
| GO:0055085 | transmembrane transport | 74 | 1.48E-2 |
| GO:0030111 | regulation of Wnt signaling pathway | 26 | 1.48E-2 |
| GO:0009891 | positive regulation of biosynthetic process | 126 | 1.49E-2 |
| GO:0051093 | negative regulation of developmental process | 69 | 1.51E-2 |
| GO:0043393 | regulation of protein binding | 20 | 1.52E-2 |
| GO:0031328 | positive regulation of cellular biosynthetic process | 124 | 1.53E-2 |
| GO:0002520 | immune system development | 56 | 1.56E-2 |
| GO:0007568 | aging | 26 | 1.57E-2 |
| GO:1901360 | organic cyclic compound metabolic process | 257 | 1.58E-2 |
| GO:0045429 | positive regulation of nitric oxide biosynthetic process | 8 | 1.62E-2 |
| GO:0070647 | protein modification by small protein conjugation or removal | 57 | 1.65E-2 |
| GO:0044257 | cellular protein catabolic process | 41 | 1.69E-2 |
| GO:0006725 | cellular aromatic compound metabolic process | 245 | 1.75E-2 |
| GO:0044712 | single-organism catabolic process | 57 | 1.92E-2 |
| GO:0048646 | anatomical structure formation involved in morphogenesis | 79 | 2,00E-02 |
| GO:0003008 | system process | 92 | 2,00E-02 |
| GO:0031327 | negative regulation of cellular biosynthetic process | 100 | 2.01E-2 |
| GO:0032269 | negative regulation of cellular protein metabolic process | 70 | 2.02E-2 |
| GO:0048514 | blood vessel morphogenesis | 38 | 2.05E-2 |
| GO:0002009 | morphogenesis of an epithelium | 44 | 2.05E-2 |
| GO:0048489 | synaptic vesicle transport | 15 | 2.06E-2 |
| GO:0097480 | establishment of synaptic vesicle localization | 15 | 2.06E-2 |
| GO:0051174 | regulation of phosphorus metabolic process | 107 | 2.08E-2 |
| GO:0061440 | kidney vasculature development | 6 | 2.11E-2 |
| GO:0061437 | renal system vasculature development | 6 | 2.11E-2 |
| GO:0043436 | oxoacid metabolic process | 62 | 2.11E-2 |
| GO:0048732 | gland development | 40 | 2.17E-2 |
| GO:0048709 | oligodendrocyte differentiation | 11 | 2.17E-2 |
| GO:0006325 | chromatin organization | 47 | 2.18E-2 |
| GO:0001654 | eye development | 35 | 2.18E-2 |
| GO:0019752 | carboxylic acid metabolic process | 58 | 2.2E-2 |
| GO:0050865 | regulation of cell activation | 39 | 2.23E-2 |

| | | | |
|---|---|---|---|
| GO:0009890 | negative regulation of biosynthetic process | 101 | 2.25E-2 |
| GO:0002768 | immune response-regulating cell surface receptor signaling pathway | 14 | 2.25E-2 |
| GO:0030217 | T cell differentiation | 18 | 2.35E-2 |
| GO:0051347 | positive regulation of transferase activity | 41 | 2.36E-2 |
| GO:0014706 | striated muscle tissue development | 30 | 2.37E-2 |
| GO:0051260 | protein homooligomerization | 26 | 2.37E-2 |
| GO:0097479 | synaptic vesicle localization | 15 | 2.45E-2 |
| GO:0046887 | positive regulation of hormone secretion | 15 | 2.45E-2 |
| GO:0019220 | regulation of phosphate metabolic process | 106 | 2.56E-2 |
| GO:0032446 | protein modification by small protein conjugation | 49 | 2.58E-2 |
| GO:0002064 | epithelial cell development | 24 | 2.59E-2 |
| GO:0006139 | nucleobase-containing compound metabolic process | 234 | 2.59E-2 |
| GO:0045935 | positive regulation of nucleobase-containing compound metabolic process | 118 | 2.62E-2 |
| GO:0009057 | macromolecule catabolic process | 55 | 2.62E-2 |
| GO:0060740 | prostate gland epithelium morphogenesis | 7 | 2.64E-2 |
| GO:0043549 | regulation of kinase activity | 56 | 2.66E-2 |
| GO:0046483 | heterocycle metabolic process | 242 | 2.67E-2 |
| GO:1903053 | regulation of extracellular matrix organization | 6 | 2.69E-2 |
| GO:0031497 | chromatin assembly | 13 | 2.71E-2 |
| GO:0009605 | response to external stimulus | 112 | 2.72E-2 |
| GO:0043065 | positive regulation of apoptotic process | 45 | 2.72E-2 |
| GO:0045892 | negative regulation of transcription, DNA-templated | 84 | 2.74E-2 |
| GO:0045860 | positive regulation of protein kinase activity | 36 | 2.74E-2 |
| GO:0008104 | protein localization | 108 | 2.79E-2 |
| GO:0045597 | positive regulation of cell differentiation | 66 | 2.8E-2 |
| GO:0007411 | axon guidance | 20 | 2.87E-2 |
| GO:0044265 | cellular macromolecule catabolic process | 50 | 2.89E-2 |
| GO:0002764 | immune response-regulating signaling pathway | 18 | 2.89E-2 |
| GO:0072073 | kidney epithelium development | 18 | 2.89E-2 |
| GO:0051223 | regulation of protein transport | 56 | 2.89E-2 |
| GO:0051603 | proteolysis involved in cellular protein catabolic process | 39 | 2.92E-2 |
| GO:0002521 | leukocyte differentiation | 31 | 2.93E-2 |
| GO:0045934 | negative regulation of nucleobase-containing compound metabolic process | 95 | 2.94E-2 |
| GO:0009896 | positive regulation of catabolic process | 36 | 2.96E-2 |
| GO:0045725 | positive regulation of glycogen biosynthetic process | 5 | 2.96E-2 |
| GO:0022612 | gland morphogenesis | 16 | 2.99E-2 |
| GO:0034109 | homotypic cell-cell adhesion | 29 | 3,00E-02 |
| GO:0006900 | membrane budding | 9 | 3.02E-2 |
| GO:0097485 | neuron projection guidance | 20 | 3.02E-2 |
| GO:0044092 | negative regulation of molecular function | 73 | 3.02E-2 |
| GO:0045930 | negative regulation of mitotic cell cycle | 19 | 3.06E-2 |
| GO:0048857 | neural nucleus development | 10 | 3.09E-2 |
| GO:0060512 | prostate gland morphogenesis | 7 | 3.13E-2 |
| GO:0060627 | regulation of vesicle-mediated transport | 35 | 3.16E-2 |
| GO:0006807 | nitrogen compound metabolic process | 279 | 3.18E-2 |
| GO:0043068 | positive regulation of programmed cell death | 45 | 3.2E-2 |

| | | | |
|---|---|---|---|
| GO:0051253 | negative regulation of RNA metabolic process | 88 | 3.25E-2 |
| GO:0043087 | regulation of GTPase activity | 42 | 3.25E-2 |
| GO:0032733 | positive regulation of interleukin-10 production | 6 | 3.33E-2 |
| GO:0034220 | ion transmembrane transport | 59 | 3.33E-2 |
| GO:0045184 | establishment of protein localization | 83 | 3.36E-2 |
| GO:0007088 | regulation of mitotic nuclear division | 15 | 3.36E-2 |
| GO:0006082 | organic acid metabolic process | 62 | 3.38E-2 |
| GO:0007519 | skeletal muscle tissue development | 17 | 3.54E-2 |
| GO:0007159 | leukocyte cell-cell adhesion | 27 | 3.54E-2 |
| GO:0008285 | negative regulation of cell proliferation | 49 | 3.65E-2 |
| GO:0001504 | neurotransmitter uptake | 4 | 3.68E-2 |
| GO:0051654 | establishment of mitochondrion localization | 4 | 3.68E-2 |
| GO:0050851 | antigen receptor-mediated signaling pathway | 11 | 3.79E-2 |
| GO:0030001 | metal ion transport | 46 | 3.79E-2 |
| GO:1903507 | negative regulation of nucleic acid-templated transcription | 84 | 3.88E-2 |
| GO:1902679 | negative regulation of RNA biosynthetic process | 85 | 3.94E-2 |
| GO:0006091 | generation of precursor metabolites and energy | 23 | 3.94E-2 |
| GO:0030238 | male sex determination | 5 | 3.94E-2 |
| GO:0070875 | positive regulation of glycogen metabolic process | 5 | 3.94E-2 |
| GO:1901881 | positive regulation of protein depolymerization | 5 | 3.94E-2 |
| GO:0044708 | single-organism behavior | 38 | 3.94E-2 |
| GO:0032479 | regulation of type I interferon production | 9 | 3.94E-2 |
| GO:0009185 | ribonucleoside diphosphate metabolic process | 9 | 3.94E-2 |
| GO:0044087 | regulation of cellular component biogenesis | 53 | 3.97E-2 |
| GO:0043010 | camera-type eye development | 31 | 3.98E-2 |
| GO:2000113 | negative regulation of cellular macromolecule biosynthetic process | 91 | 4.02E-2 |
| GO:0050770 | regulation of axonogenesis | 16 | 4.02E-2 |
| GO:0051172 | negative regulation of nitrogen compound metabolic process | 100 | 4.02E-2 |
| GO:2000772 | regulation of cellular senescence | 6 | 4.1E-2 |
| GO:2000826 | regulation of heart morphogenesis | 6 | 4.1E-2 |
| GO:0014066 | regulation of phosphatidylinositol 3-kinase signaling | 11 | 4.16E-2 |
| GO:0044281 | small molecule metabolic process | 98 | 4.22E-2 |
| GO:0002429 | immune response-activating cell surface receptor signaling pathway | 12 | 4.25E-2 |
| GO:1903428 | positive regulation of reactive oxygen species biosynthetic process | 8 | 4.3E-2 |
| GO:0006836 | neurotransmitter transport | 16 | 4.33E-2 |
| GO:0016567 | protein ubiquitination | 45 | 4.33E-2 |
| GO:0032410 | negative regulation of transporter activity | 9 | 4.48E-2 |
| GO:0090304 | nucleic acid metabolic process | 208 | 4.5E-2 |
| GO:0070201 | regulation of establishment of protein localization | 58 | 4.52E-2 |
| GO:0033674 | positive regulation of kinase activity | 37 | 4.57E-2 |
| GO:0043161 | proteasome-mediated ubiquitin-dependent protein catabolic process | 25 | 4.59E-2 |
| GO:0010634 | positive regulation of epithelial cell migration | 13 | 4.6E-2 |
| GO:0001959 | regulation of cytokine-mediated signaling pathway | 11 | 4.6E-2 |
| GO:0009132 | nucleoside diphosphate metabolic process | 11 | 4.6E-2 |
| GO:0015711 | organic anion transport | 28 | 4.62E-2 |
| GO:0030178 | negative regulation of Wnt signaling pathway | 16 | 4.64E-2 |

| | | | |
|---|---|---|---|
| GO:0044255 | cellular lipid metabolic process | 55 | 4.66E-2 |
| GO:0010942 | positive regulation of cell death | 46 | 4.9E-2 |
| GO:0016070 | RNA metabolic process | 184 | 4.94E-2 |
| GO:0033059 | cellular pigmentation | 8 | 4.96E-2 |

**Supplementary Table S2.** List of molecular interactions leading miR-125a-3p to inhibit MBP expression.

| Molecule A | Molecule B | Type of interaction | Function | Reference |
|---|---|---|---|---|
| miR-125a-3p | FYN | Binding | Inhibition of Fyn expression | Ninio-Many et al., 2013 |
| miR-125a-3p | SMAD4 | Binding | Inhibition of Smad4 expression | Boissart et al., 2012 |
| miR-125a-3p | RHOA | Binding | Inhibition of RhoA expression | Huang et al., 2013 |
| miR-125a-3p | P38 | Binding | Inhibition of p38 expression | Dong et al., 2014 |
| miR-125a-3p | NRG-1 | Binding | Inhibition of NRG-1 expression | Yin et al., 2015 |
| FYN | QKI | Phosphorylation | Activation of QKI | Lu et al., 2005 |
| FYN | MBP | Indirect mechanism | Stimulation of MBP promoter | Umemori et al., 1999 |
| FYN | RHOA | Phosphorylation | Inhibition of RHOA via p190RhoGAP activation | Wolf t al., 2001; Liang et al., 2004 |
| QKI | MAP1B | Binding | Stabilization of MAP1B mRNA | Zhao et al., 2006 |
| QKI | MBP | Binding | Stabilization of MBP mRNA | Li et al., 2000 |
| NRG-1 | MBP | Indirect mechanism | Promotion of MBP expression | Cannella et al., 1998 |
| NRG-1 | FYN | Indirect mechanism | Binding of NRG-1 to ErbB4 increases Fyn activation | Bjarnadottir et al., 2007 |
| RHOA | P38 | Indirect mechanism | Promotion of p38 activation | Ivaska et al., 1999 |
| SMAD4 | P38 | Indirect mechanism | Activation of P38 via MAPKKK | Takekawa et al., 2008 |
| P38 | MBP | Phosphorylation | Promotes compaction of myelin | Unpublished observation |

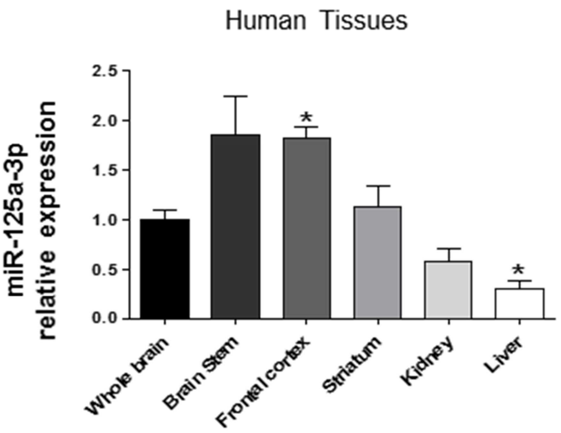


**Supplementary Figure S1.** miR-125a-3p relative expression was analyzed using Taqman assay in different human tissues and compared to level measured in whole brain. Neural tissues showed higher expression with respect to other tissues. Data were expressed as mean ± SEM, n = 3; two-tailed unpaired t test *p < 0.05 vs. whole brain.

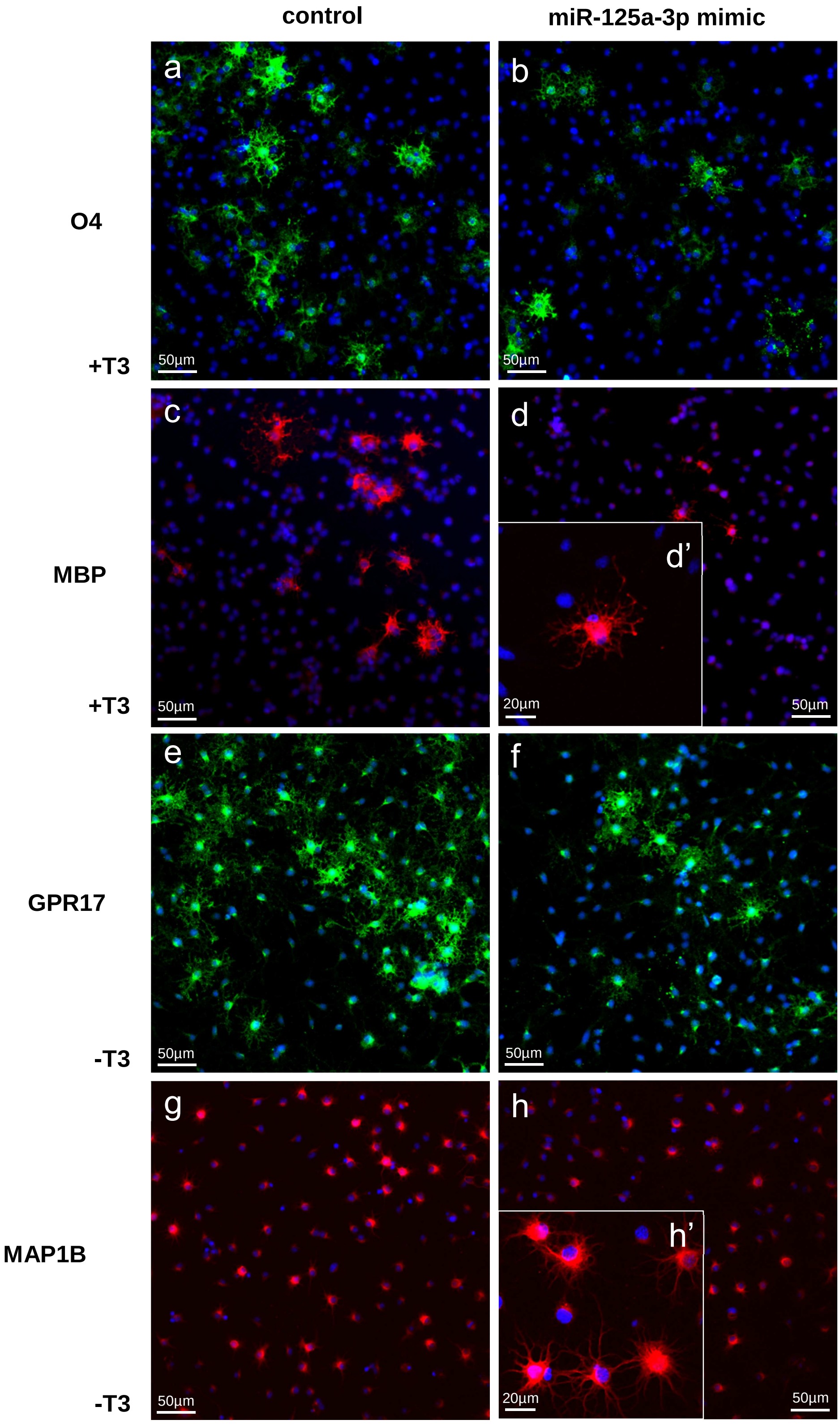


**Supplementary Figure S2.** Representative micrographs of OPC cultures 48h after transfection with miR-125a-3p mimic compared to control cultures, at both low (a-h) and high magnification (d', h'). After culturing in a standard proliferation medium, OPCs were transfected and differentiated either in the presence (a-d) or in the absence (e-h) of the T3 hormone, according to a "fast" and a "slow differentiation protocol", respectively. Then, cells were fixed and stained for the selected markers: (a,b) O4, (c,d,d') MBP, (e,f) GPR17, (g,h,h') MAP1B.

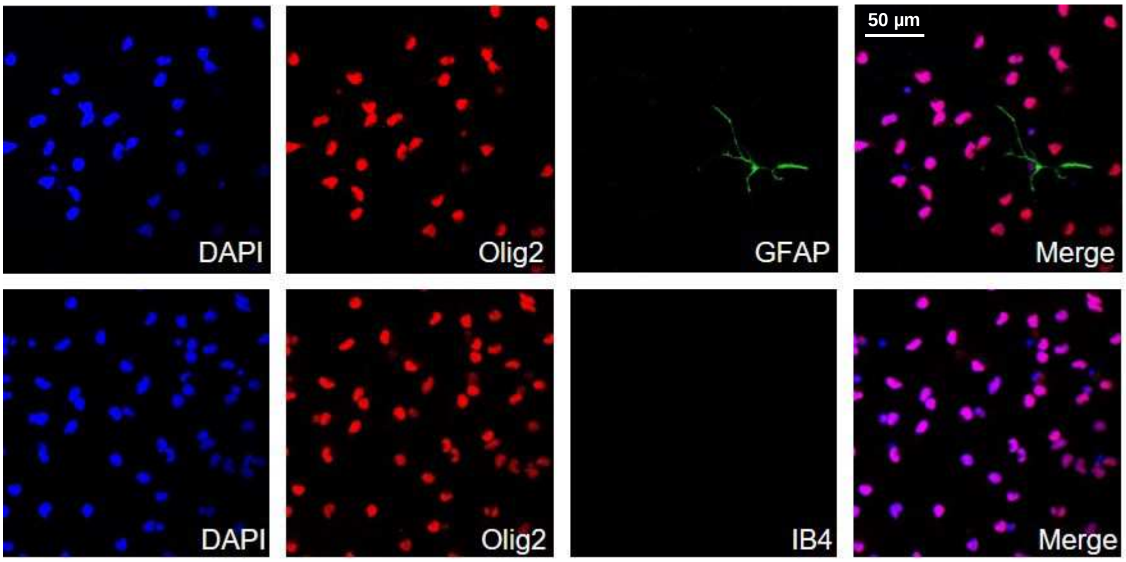


**Supplementary Figure S3.** Representative images of OPC cultures to investigate the potential contamination by other cell types. After culturing in a standard proliferation medium, OPCs were differentiated for 48 hours in the presence of the T3 hormone. Then, cells were fixed and stained for the selected markers: Olig2 for oligodendrocytes, GFAP for astrocytes and Ib4 for microglia cells.
